# Effects of a 30 K Military Loaded Carriage on the Neuromuscular System in Spanish Army Marines

**DOI:** 10.3390/sports13030076

**Published:** 2025-03-05

**Authors:** Beltrán Cáceres-Diego, Cristian Marín-Pagán, Pablo Martínez de Baños, Pedro E. Alcaraz

**Affiliations:** 1 UCAM Research Center for High Performance Sports, UCAM Universidad Católica de Murcia, 30107 Murcia, Spain; cmarin@ucam.edu (C.M.-P.); palcaraz@ucam.edu (P.E.A.); 2 Strength and Conditioning Society, 30008 Murcia, Spain; 3 Department of Instruction and Training, Marine Infantry School “General Albacete y Fuster”, 30201 Cartagena, Spain; pmarma9@mde.es; 4 Facultad de Deporte, UCAM Universidad Católica de Murcia, 30107 Murcia, Spain

**Keywords:** load carriage, neuromuscular fatigue, performance, soldier, physical readiness and conditioning

## Abstract

Infantry soldiers must cover long distances carrying heavy and bulky combat equipment. Since the beginning of their training, Spanish Marines have undergone this characteristic and demanding test. However, little is known about its effects on neuromuscular function and recovery in the days following the test. Twenty-six Spanish Marines completed the test, three of whom suffered injuries and had to withdraw from the study, resulting in a final sample of twenty-three Marines. These participants underwent evaluations before (pre), immediately after (post), and 24 and 48 h post-exercise, following a 30 km endurance march carrying their 34 kg combat equipment. A repeated-measures ANOVA, paired-samples *t*-test, and effect size (ES) analysis were conducted; the results are presented as mean ± SD. The significance level was set at *p* ≤ 0.05. The variables and *p*-values of changes over time are presented. Isometric mid-thigh pull (IMTP) (*p* = 0.004), countermovement jump (CMJ) (*p* ≤ 0.001), rating of fatigue scale (ROF) (*p* ≤ 0.001), maximum pull-ups in two minutes (PUmax) (*p* ≤ 0.001), body mass (BM) (*p* ≤ 0.001), hand grip strength (HGS): dominant (*p* = 0.180) and non-dominant (*p* = 0.616), and incident reports (IRPE) showed a significant increase over time and between the first 10 km and last 5 km in fatigue, muscle pain, joint pain, shortness of breath, excessive sweating (*p* ≤ 0.001), and muscle tremors (*p* = 0.028), except for palpitations (*p* = 0.189). In conclusion, the results indicate that the test had a significant impact on neuromuscular function, with no recovery observed in overall strength and lower limb power after 48 h, even though their perceived fatigue decreased substantially. The resilient spirit of operational military units and their philosophy of always being ready for combat could increase the injury rate.

## 1. Introduction

The mission of the Army is to ensure national defense by safeguarding its territory, protecting its citizens, and upholding constitutional order. To fulfill these duties, military personnel must undergo rigorous physical and mental preparation to guarantee success when the duty calls. In this preparation, military maneuvers or exercises play a central role, serving to teach and assess the skills of both the unit and its members. Specifically, infantry soldiers are characterized by covering long distances on foot (10–20 km) while carrying bulky and heavy equipment (15–61 kg) [1,2,3], across rough terrain inaccessible to vehicles. The success of the mission or survival may depend on how quickly a soldier can move from one point to another while carrying personal combat gear, tactical equipment, and/or supplies [4]. For this reason, loaded marches are one of the primary tasks performed by military personnel during their training maneuvers, which place a high physical demand on them [5].

This physical demand is determined by the load carried, an individual’s body mass, load distribution, movement speed, body armor, and the type of terrain [1,6,7]. Among these factors affecting performance in this task, particular attention is given to load distribution, the carried weight, and physical condition. For each kilogram of added load, military task performance decreases by 1–3% due to increased aerobic and muscular strength demands [6,8]. Positioning the load close to the soldier’s center of mass, over the torso and shoulder girdle, results in the lowest energy expenditure [1]. Furthermore, poor physical condition can lead to the deformation of soft tissues [9,10], which impairs respiratory mechanics, imposes additional physiological stress, and alters movement biomechanics [11], all of which negatively affect work capacity [6,7]. This may affect neuromuscular function, influencing the interaction between the nervous system and muscles, potentially compromising the muscle’s contractile capacity to generate force and move efficiently. This, in turn, impacts operational performance and subsequent recovery.

In this regard, musculoskeletal injuries are prevalent and appear to be associated with the demands encountered by military instruction, training, and preparation. These injuries are among the leading causes of attrition, reduced productivity, and suboptimal military readiness, resulting in significant economic and human costs due to medical care and the inability to perform tasks effectively [12]. Among the wide range of injuries prevalent in the Army, most are related to training, specifically due to overuse or excessive strain, indicating that soldiers are overtrained [13]. These injuries greatly impair military strength and operational capability and are defined as injuries that occur gradually as a result of high levels of mechanical stress without sufficient time for recovery, structural repair, and metabolic adaptation. They are caused by accumulated microtraumas resulting from repetitive and intense movement patterns [14,15], with significantly higher incidence rates among women [16].

Primarily for infantry soldiers, these types of military practices are often repeated over several consecutive days or conducted alongside other physically demanding military tasks, with little to no rest, in a stressful environment where sleeping and eating are challenging. Therefore, this characteristic military task, the loaded march, could impact the effectiveness of performing other duties in the following days due to its high physical demands. For this reason, analyzing these common practices to observe their impact on the body, as well as the recovery process in the subsequent days, is of great importance for both physical and military preparation.

Previous research on the physiological and biomechanical performance of military personnel during long-distance running while carrying a load, such as combat equipment, has demonstrated significant effects on muscle fatigue, energy expenditure, and injury risk [1,3,17]. Studies [6,18,19] have shown that carrying heavy loads during prolonged exertion alters running economy, increasing metabolic cost and reducing biomechanical efficiency due to changes in gait kinematics and impact mechanics—factors that may compromise operational performance and recovery, such as increased knee and hip flexion, forward trunk inclination, and a slight tendency to take fewer strides per unit of time as the carried load increases. However, there is a notable lack of studies that comprehensively analyze the combined effects of load, distance, and terrain on performance and physiological resilience in operational contexts. Most previous studies [20,21,22] have focused on shorter distances or laboratory tests under controlled conditions, leaving a gap in understanding the impact of these demands in real-world deployment scenarios. This gap in the literature underscores the need for the present study, which aims to address these limitations through a more specific evaluation of physiological responses in longer-duration tests under operational conditions. In doing so, it seeks to provide key insights into the neuromuscular fatigue induced by such exertion and the subsequent recovery process, ultimately contributing to the optimization of military training and preparedness. Data collected in this research could help optimize training loads to promote recovery and consolidate physiological adaptations after assessing the march’s impact. Additionally, it could aid in designing strategies to improve performance and recovery after the march, as it is a mandatory test in the training and instruction program of the Spanish Marine Infantry. Therefore, the objective of this study was to analyze and describe the neuromuscular fatigue caused by a 30 km conditioning military march with combat gear, as well as the recovery process.

## 2. Materials and Methods

### 2.1. Study Design

A descriptive repeated-measures study was conducted on a 30 km conditioning march with 34 kg of combat logistical gear over a 5 km circuit with elevation changes, initially involving 26 military personnel. Evaluations were conducted pre-march, post-march, 24 h post-march, and 48 h post-march. The total distance traveled and elevation gain, along with other variables, were quantified using identical smart sports watches equipped with global positioning system (GPS) technology. The collected data were expressed as mean ± SD. The logistical combat gear weighed 34 kg and consisted of a 48–72 h survival backpack, helmet, rifle, boots, and a MOLLE system plate carrier with included magazine pouches. After familiarization with performance tests and 24 h of rest from vigorous exercise, participants performed a general warm-up followed by a specific warm-up, after which baseline data (pre-training) were collected, including age, height, and body mass (BM). The activity or test conducted was an official maneuver organized by the military academy, with its planning and workload specifically adjusted for the execution on that day. This approach ensured that the soldiers were optimally prepared and mentally committed to achieving peak performance, as the test was an official, graded component of the academy’s program, rather than an additional external evaluation. Additionally, the following performance tests were conducted in the specified order, with 3–5 min of rest between each: countermovement jump (CMJ), isometric mid-thigh pull (IMTP), hand grip strength (HGS), rate of perceived exertion scale (ROF), and a maximum pull-up test in 2 min (PUmax). After the 30 km march with combat gear, data collection, and performance tests were repeated post-march to assess acute fatigue induced by the activity. Subsequent evaluations were conducted at 24 and 48 h post-march to observe residual fatigue. Finally, an incident report was completed at the end of the test.

### 2.2. Participants

The initial sample consisted of 26 male military personnel from the Spanish Army Marine Infantry School. During the march, three participants sustained injuries related to knee and lower back issues and had to withdraw, resulting in a final sample of 23 participants (age: 28.4 ± 5.0 years; height: 176.2 ± 5.8 cm; body mass: 80.0 ± 7.8 kg). The inclusion criteria were as follows: (a) being active military personnel, (b) belonging to the CAES (Access Course to the Non-Commissioned Officer Scale) of the Marine Infantry Corps, and (c) not suffering from any disease, injury, or deficiency of any kind. The exclusion criteria were as follows: (a) having a diagnosed illness, injury, or deficiency that could affect physical performance during the study, (b) undergoing pharmacological treatment that could influence physical performance, (c) failing to complete all phases and protocols of the study, and (d) voluntarily expressing the desire to withdraw from the study at any time. The sample consisted exclusively of men, as no women were present in the CAES at the time of the study. Before the study began, participants were informed of the details of the intervention and signed an informed consent document in accordance with the Declaration of Helsinki [23]. All procedures were approved by the Ethics Committee of the Catholic University of Murcia, UCAM (28 October 2022, CE102202). The participants were recruited through a collaboration agreement between the Ministry of Defense of the Kingdom of Spain and UCAM (BOE-A-2022-12703).

### 2.3. Procedures

Warm-up. The warm-up performed by participants before each data collection consisted of the following: (a) joint mobility exercises, with 1 set of 10 repetitions for each of the following exercises: shoulder rotations, trunk rotations, hip twists, and standing knee flexions forward; (b) dynamic stretches, with 1 set of 10 repetitions for each of the following exercises: standing hip flexion–extension reaching toward ground, standing hamstring stretch lifting leg to horizontal and touching toe with hand; (c) metabolic exercises, with 1 set of 8–20 repetitions for each of the following exercises: jumping jacks, skipping, lunges, and burpees; and (d) a specific exercise, the straight bar deadlift: set 1 (4 × 20 kg), set 2 (6 × 40 kg), and set 3 (4 × 60 kg) with 1–2 min of rest between sets.

Maximum Isometric Leg and Back Strength. The isometric mid-thigh pull (IMTP) is a valid and reliable test for assessing an individual’s overall maximum strength [24]. Participants performed the test on a Smith machine placed on a portable force platform (Kistler 9286BA, Kistler Group, Winterthur, Switzerland). The barbell was fixed and adjusted to ensure an equal distance between the greater trochanter and the lateral epicondyle of both knees. Participants first completed a submaximal practice trial, which lasted between 3 and 5 s. Subsequently, participants performed 2 maximal repetitions, each lasting between 3 and 5 s, with 20 s of rest between repetitions. The best result was used for analysis.

Countermovement jump (CMJ). The CMJ is the most validated, reliable, and widely used test for estimating lower-limb power strength [25]. The CMJ was performed on a portable force platform capable of performing stabilometric analysis via the center of pressure oscillations using four piezoelectric sensors, located at each end of the platform, with a range of 400–600 mm. The sampling frequency was 1000 Hz, allowing for the recording of displacement and contact time between the foot and the platform surface to be recorded. Data were recorded and analyzed using Kistler’s measurement, analysis, and reporting software (MARS, 2012 S2P Ltd., Ljubljana, Slovenia), because most of the squat parameters and data obtained from MARS are consistent, reliable, and suitable for monitoring individuals as well as for quantifying variations in performance [26]. Raw data were exported, and maximum vertical jump height from take-off (VJ) [cm], peak power (PP) [W], peak eccentric force (PEcc), concentric force (PCon) [N], and rate of force development in the eccentric (RFDEcc) and concentric phases (RFDCon) [N/s], were calculated using Microsoft Excel (Microsoft Corporation, Redmond, DC, USA). The depth of countermovement was self-selected by participants, who were instructed to land close to the take-off point. Participants performed two jumps with a 60 s rest between them, and the best jump was selected based on the maximum height achieved.

Rating of fatigue scale (ROF). Athletes were asked to rate their perceived level of fatigue after completing the test using a numerical scale from 0 to 10, where 0 represents a state of rest, and 10 represents maximal effort [27]. This numerical value was used to define fatigue or the subjective effort experienced by the individual following exertion.

Maximal pull-ups in 2 min (PUmax). This test, used to measure muscular strength and/or endurance in military personnel [5,28], involved lifting one’s own body mass as many times as possible, with the chin clearing a horizontal bar. The bar was gripped in a pronated position with a width equal to or slightly greater than shoulder width. The starting position was hanging from the bar with elbows extended and feet off the ground, and the final position required the elbows to be bent, and the body to be elevated until the chin was above the bar with the Adam’s apple passing the top of the bar [29,30]. The test concluded either upon reaching exhaustion or after 2 min, during which the participant had to always maintain a grip on the bar without dropping or resting on any surface. Participants were informed that incorrectly performed repetitions with poor technique would not be counted, and a brief pause of less than 10 s was permitted. The maximum number of correctly executed repetitions was recorded for analysis.

Hand grip strength (HGS). HGS is an essential test for assessing performance in manual lifting and carrying tasks [31]. To measure hand grip strength, a Camry digital hand dynamometer (model EH101, Zhongshan Camry Electronic Co., Ltd., China) was used. The shoulder of the testing arm was kept close to the side with the elbow at 90° and the grip in a neutral position. Two repetitions were performed for each hand, dominant (DHGS) and non-dominant (NDHGS), applying maximal force for 3 s with a 60 s rest between repetitions. The best attempt was used for the analysis.

Incident Reports and Perceived Effort (IRPE) during the test. Incidences, discomfort, symptoms, and perceived effort during the test were recorded for each participant using a report form. Participants noted their subjective feelings of fatigue, muscle pain, joint pain, shortness of breath, palpitations, excessive sweating, and muscle tremors on a scale with four ranges (0: none, 30: slight, 60: moderate, and 90: severe) at various segments of the test (0–10 km, 10–15 km, 15–20 km, 20–25 km, and 25–30 km), as well as in a section for observations where they recorded any issues encountered upon completing the 30 km test.

### 2.4. Statistical Analyses

Statistical analysis was conducted using Jamovi software version 1.6 (The Jamovi Project, Sydney, Australia). Inferences were calculated based on the magnitude of fluctuations in performance variables pre-test, post-test, 24 h post-test, and 48 h post-test, and were presented as standardized mean differences (mean ± standard deviation, SD) [32]. The normality of the data was assessed, confirming that all analyzed variables met the established criteria. The Shapiro–Wilk test was applied for this purpose, considering both the *p*-value and the W statistic. To evaluate results over time, a repeated-measures analysis of variance (Repeated Measures ANOVA) was performed with multiple comparisons between different time points. Subsequently, each studied variable was compared across different time points of evaluation using a paired-sample *t*-test. The significance level was set at *p* ≤ 0.05. Effect size (ES) was calculated using Cohen’s d [33] by comparing pre-post, pre-24 h post, and pre-48 h post variables. This helped determine the level of significance of the effect for each studied variable, reflecting significant differences in the same variable between different time points. Thus, effect size (ES) was interpreted using Cohen’s d as follows: trivial (<0.2), small (≥0.2–<0.6), moderate (≥0.6–<1.2), large (≥1.2–<2.0), very large (≥2.0–<4.0), and extremely large (≥4.0) [34]. In summary, the recommendations on the use of statistics in sports medicine and exercise sciences were followed [34].

## 3. Results

Regarding march characteristics collected by similar smart sports watches from 12 participants, data are presented as mean ± SD. However, the total distance of the test was initially measured by the supervising and command team to ensure accuracy. The total distance was 29.98 ± 0.34 km. The total time was 327.67 ± 17.37 min and 34.58 ± 17.17 s. The average pace was 10.95 ± 0.5 min/km. The total estimated calories recorded were 2382.0 ± 301.83 kcal, calculated based on the individual’s anthropometric characteristics, physical condition, and the activity performed, such as age, height, body mass, sex, type of activity, duration, intensity, and heart rate during the test. Average heart rate was 131.75 ± 10.59 bpm, with peak values reaching 173.25 ± 11.63 bpm. The average temperature during the test was 21.4 ± 2.56 °C, with maximum temperatures of 24.2 ± 2.18 °C. Total ascent was 628.25 ± 426.47 m, and total descent was 859.75 ± 752.93 m. The average speed was 5.51 ± 0.28 km/h, with maximum speeds reaching 11.52 ± 2.21 km/h. The total time for 23 participants who completed the test was 340.36 ± 17.70 min.

**Figure A1:**
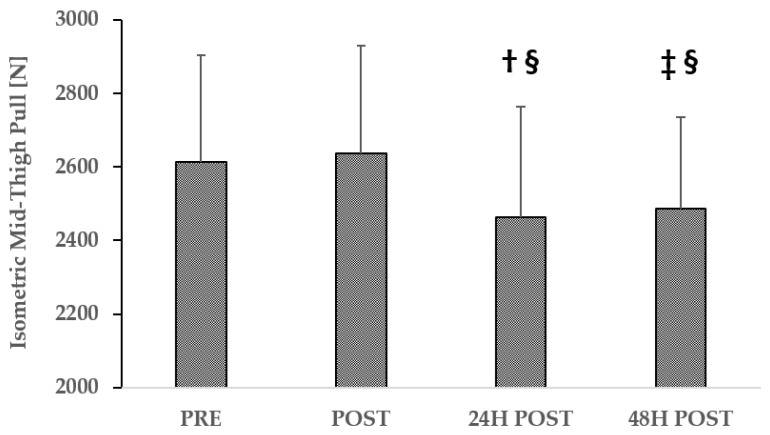
Evolution of results for mid-thigh isometric pull test. Values are represented as means ± SD. The following symbols indicate significant differences relative to (a) Pre: † = significant difference (*p* ≤ 0.01); ‡ = significant difference (*p* ≤ 0.001); and (b) Post: § = significant difference (*p* ≤ 0.05).

The results for performance variables are presented in Table A1 (see Appendix A.1). Peak absolute vertical strength, measured by the isometric mid-thigh pull (IMTP) test, showed a significant decrease over time (*p* = 0.004) (see Figure A1, Appendix A.2). There were no significant changes (0.98%) post-test compared to pre-test values, but there was a significant decrease (*p* = 0.037) at 24 h post-test (−5.7%) and at 48 h post-test (−4.8%) compared to pre-test values (*p* = 0.005).

**Figure A2:**
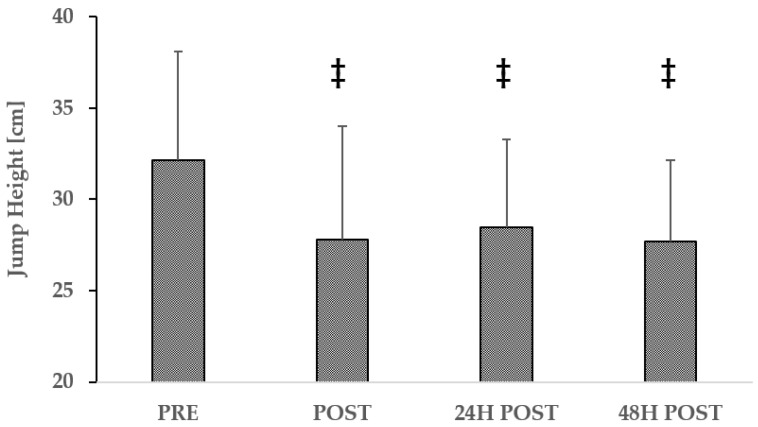
Evolution of results for countermovement jump assessment test. Values are represented as means ± SD. The following symbol indicates significant differences relative to Pre: ‡ = significant difference (*p* ≤ 0.001).

Regarding countermovement jump (CMJ) variables, a significant decrease over time was found in vertical jump height (VJ) (*p* < 0.001), peak power (PP) (*p* < 0.001), concentric rate of force development (RFDCon) (*p* = 0.033), and peak eccentric force (PEcc) (*p* = 0.012). However, there were no significant changes in peak concentric force (PCon) (*p* = 0.190) or in the eccentric rate of force development (RFDEcc) (*p* = 0.487). The evolution of changes at different time points varied across CMJ variables: VJ showed a very pronounced decrease pre-post (−13.7%), with similar decreases 24 h post (−11.5%) and 48 h post (−14.0%) compared to pre-test values (see Figure A2, Appendix A.3). PP exhibited no significant change pre-post (−1.0%), but there was a significant decrease pre-24 h (−8.6%) and pre-48 h (−9.1%). RFDCon showed no significant changes pre-post (6.5%), with a substantial decrease of (−46.0%) at 24 h and (−12.0%) at 48 h compared to pre-test values. PEcc highlighted a decrease (−6.9%) pre-post, with a quick recovery (−0.4%) pre-24 h, and even an increase (0.4%) pre-48 h. PCon showed no significant changes (1.7%) pre-post, with decreases of (−2.3%) pre-24 h and (0.3%) pre-48 h. RFDEcc exhibited no significant changes (−4.4%) pre-post, with increases in (6.5%) pre-24 h and (3.5%) pre-48 h.

**Figure A3:**
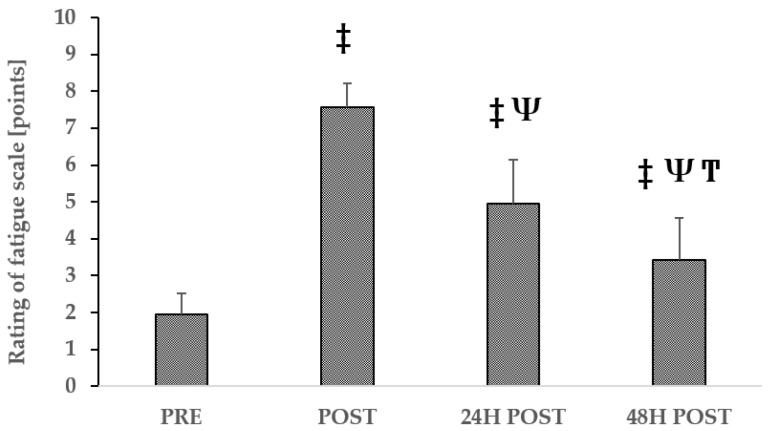
Evolution of results for rating of fatigue scales test. Values are represented as means ± SD. The following symbols indicate significant differences relative to (a) Pre: ‡ = significant difference (*p* ≤ 0.001); (b) Post: Ψ = significant difference (*p* ≤ 0.001); and (c) 24 h post: Ͳ = very significant difference (*p* ≤ 0.001).

**Figure A4:**
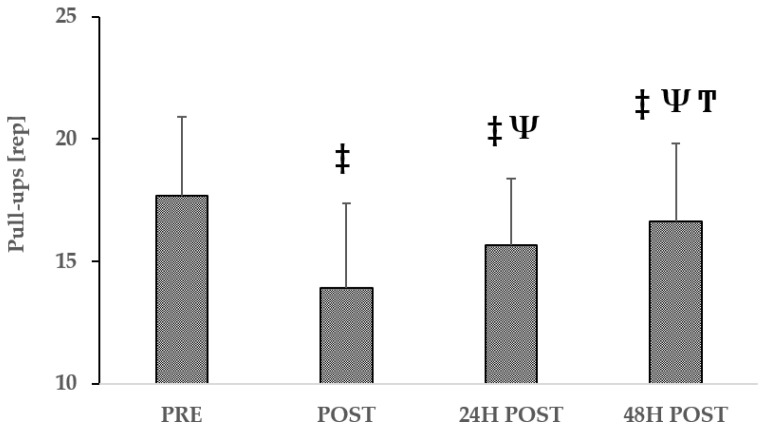
Evolution of results for pull-ups test. Values are represented as means ± SD. The following symbols indicate significant differences relative to (a) Pre: ‡ = significant difference (*p* ≤ 0.001); (b) Post: Ψ = significant difference (*p* ≤ 0.001); and (c) 24 h post: Ͳ = very significant difference (*p* ≤ 0.001).

**Figure A5:**
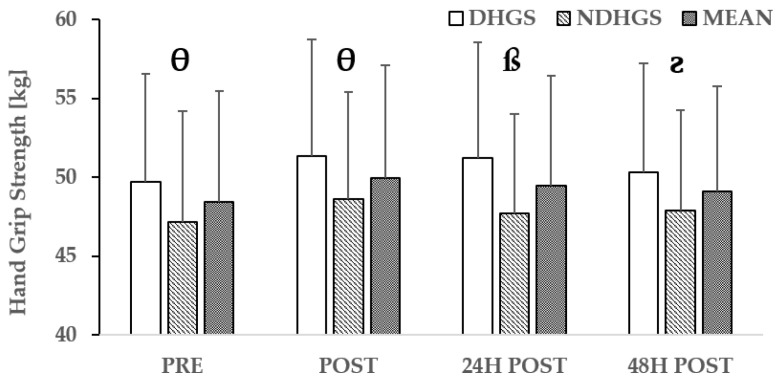
Evolution of results for hand grip strength assessment test. Values are represented as means ± SD. The following symbols indicate significant differences between DHGS and NDHGS: ƨ = significant difference (*p* ≤ 0.05); θ = significant difference (*p* ≤ 0.01); ß = significant difference (*p* ≤ 0.001).

Rating of fatigue (ROF) exhibited significant changes (*p* < 0.001) across different time points, showing increases in pre-post (286.2%), pre-24 h (153.1%), and pre-48 h (75.0%) (see Figure A3, Appendix A.4). The pull-up test (PUmax) demonstrated significant changes over time (*p* < 0.001), with decreases in pre-post (−21.5%), pre-24 h (−11.3%), and pre-48 h (−5.6%) (see Figure A4, Appendix A.5). All body mass (BM) evaluations showed a decrease (*p* < 0.001) following the test: pre-post (−3.4%); pre-24 h (−2.0%); and pre-48 h (−1.9%). The hand grip strength test (HGS) did not show significant changes between different time points overall (*p* = 0.343), including in the dominant hand (DHGS) (*p* = 0.180) and non-dominant hand (NDHGS) (*p* = 0.616). However, a slight increase was observed in DHGS of +3.24% (*p* = 0.054) pre-post, +3.02% (*p* = 0.108) pre-24 h, and +1.19% (*p* = 0.525) pre-48 h; and in NDHGS of +3.18% (*p* = 0.301) pre-post, +1.19% (*p* = 0.563) pre-24 h, and +1.59% (*p* = 0.566) pre-48 h (see Figure A5, Appendix A.6). Additionally, significant differences were observed between the DHGS and NDHGS (*p* < 0.001), with pre-test differences in (*p* = 0.009) −5.4%, post-test (*p* = 0.004) −5.5%, post-24 h (*p* = 0.001) −7.3%, and post-48 h (*p* = 0.025) −5.0%.

Data from reports are summarized in Table A2 (see Appendix A.7). Overall, the number and severity of symptoms and incidents increased as the distance covered in kilometers increased. Significant changes (*p* < 0.001) were observed, with an increase in perceived fatigue, muscle pain, joint pain, shortness of breath, and excessive sweating across different segments of the test, as well as in muscle tremors (*p* = 0.028), with the exception of palpitations (*p* = 0.189).

Incidents reported after the test that impeded its performance generally included fatigue and insufficient recovery prior to the test, numerous cases of muscle strain and pain in the soles of the feet, quadriceps, trapezius, lower back, and calves, as well as pain in joints such as knees and shoulders, and blisters.

## 4. Discussion

In order to observe new findings on neuromuscular fatigue and recovery in the days following a prolonged load carriage test under operational military conditions, the aim of this study was to analyze and describe neuromuscular fatigue and subsequent recovery over the following days induced by a 30 km military conditioning march with 34 kg of combat gear. The primary finding was that the test caused significant fatigue in soldiers, who did not recover their overall absolute strength and maximum lower body power within 48 h, despite a substantial decrease in their perceived fatigue.

After completing the 30 km march with 34 kg of combat gear, IMTP showed an increase following the test. It has been demonstrated that central nervous system (CNS) activation increases after performing simulated military tasks [35]; thus, the test may have stimulated the neuroendocrine system, leading to values exceeding baseline levels [36]. Other studies [37,38] have observed a heightened psychophysiological response caused by stress in simulated combat tests, resulting in greater activation of the sympathetic nervous system, which leads to the release of catecholamines into the bloodstream, thereby increasing muscle strength, heart rate, and blood pressure. Despite this, the total force produced showed a significant decrease at 24 and 48 h; this may be due to a reduction in CNS activation as the body rested and returned to homeostasis. Although force levels increased post-test, special attention should be given when performing other exercises or tasks. Physiologically, prolonged, repetitive, and intense activities initiate a process starting with acute inflammation due to micro-damage to tissues involved in the activity (including muscles, nerves, tendons, and bones) proportional to dose–response [39,40]. If the activity continues in the same manner, the likelihood of developing an overuse injury increases considerably, leading to chronic inflammation and fibrosis of the neuromuscular system, which can result in suboptimal structural-tissue function and operational performance [41].

The drastic impact on lower body power, diminished after this demanding test, was reflected in significantly reduced results for the maximum vertical height reached in the CMJ at various time points (post, 24 h post, and 48 h post). In contrast to our findings, one study observed that after a 20 km march carrying an additional 46 kg, vertical jump height did not show a significant decrease [42]. Moreover, another study [2] involving a 10 km run with different loads (10, 27, and 36 kg) found that VJ and PP were higher after the test compared to baseline values. Nevertheless, data from the present study are similar to those observed in U.S. Rangers after 8 weeks of military training [43], where vertical jump height decreased by approximately 16%, lower body power by around 21%, and total dynamic lifting strength by about 20%. Our findings were also consistent with those observed in a study [44] after a ≈ 30 km run without additional load, showing a decrease of 9.8 ± 7.1% in vertical jump height. A similar trend was seen in PP, which slightly decreased after the test, followed by a more pronounced drop at 24 h, continuing into 48 h with no apparent improvement. Therefore, our data suggest that rest should be proportional to the effort exerted, allowing sufficient recovery between military activities whenever possible.

PEcc and RFDEcc obtained in the CMJ decreased after the test but showed a rapid recovery at 24 h, with values exceeding baseline levels at 48 h. Similarly, PCon and RFDCon increased immediately after the test, then decreased at 24 h, approaching baseline values at 48 h. It is well known that the application of concentric and eccentric force during running, with eccentric force being applied to a greater extent, is directly correlated with body mass (including extra load carried) and running speed [45]. This can lead to microtrauma in the contractile machinery of muscles predominantly used in running, thereby limiting their force production capacity [46]. Nevertheless, it is possible that numerous eccentric actions helped absorb energy from each impact and utilize it during the concentric propulsive phase of the stretch-shortening cycle, whose function is to enhance performance during the concentric propulsive phase, as previously observed [47]. Despite recovery and supercompensation in the following days, caution should be exercised before introducing another stimulus through training, as it may be counterproductive, especially during field maneuvers, where the body is subjected to additional stressors such as lack of sleep, food, and so on.

Despite everything, the process by which post-test IMTP values showed an increase compared to baseline, while CMJ values were significantly lower, remains unclear. If there had indeed been central nervous system activation, it should have been observed in both tests. It is possible that, although the upper body had to support weight throughout the test through isometric actions, it was the lower body that was responsible for moving the participant’s mass and additional load. Therefore, it is believed that peripheral fatigue due to numerous concentric and eccentric actions [48], as well as various functional alterations resulting from the demanding long-duration test [49], may have been greater in the lower body than in the upper body, affecting the power results observed in the CMJ.

On the other hand, the Spanish Marine Corps is accustomed to this type of highly demanding endurance tests, both physically and psychologically, which are common in their line of work. In this context, the perception of fatigue after the hardening military march, as represented by ROF, was consistent with results observed in neuromuscular tests, showing an increase of 286.2% compared to baseline values and remaining 75% higher at 48 h. This was also reflected in the performance trends of PUmax, where performance decreased immediately after the test and gradually recovered at 24 and 48 h. Furthermore, BM remained significantly reduced 48 h post-test, indicating signs of dehydration that may have hindered recovery in the days following the test. Other authors [3], who studied a 20 km march with different loads (34, 48, and 61 kg), observed that perceived intensity was higher with 34 kg compared to carrying 48 and 61 kg, with significantly lower performance in obstacle course tests and cognitive tasks. In light of the aforementioned, ROF is an indicator that, although subjective, is very simple and quick to obtain, and highly recommended for use, as it provides insight into the level of fatigue the individual is experiencing. This allows for adjustments to the next training session or preparation for the next military task or activity. Additionally, better post-test recovery strategies should be considered and implemented if the goal is to fully recover as quickly as possible to perform the next activity in optimal conditions.

The results observed in HGS show an increase in values after the test. Similar studies did not observe significant changes after a 45 min loaded march with 40% of body mass [50]. However, the increase in grip strength observed in the present study is not unprecedented [51,52] and may be due to neuromuscular adaptation resulting from familiarity with this type of test, which mitigates brachial plexus compression issues caused by carrying combat equipment on shoulders, as observed in previous studies [53,54]. Additionally, elevated catecholamine levels may have contributed to the increase in grip strength following the highly demanding march [3]. Regarding differences in grip strength between both hands, a slight bilateral deficit was observed in our study. If this deficit exceeds 10–15% [55], it could negatively impact the performance of specific military tasks that require the development of absolute strength [56], such as carrying heavy equipment, and ammunition, or simulating casualty evacuation [57]. Therefore, incorporating unilateral strength exercises into training sessions would be a viable solution to reduce this bilateral deficit and balance strength between both hands.

Finally, data collected from reports showed an increase in study variables proportional to the duration and kilometers covered in the test. It has been demonstrated that with greater duration and distance in loaded running, the risk of muscle injury or tissue overload increases due to possible changes in the contractile structures of muscles predominantly used in such activity [58]. These results allow the unit’s commanding officer to assess the workload tolerated and identify potential deficits in specific aspects of the soldier’s physical condition, enabling the design of training sessions to improve performance in particular segments of the test. In this regard, performing specific training for load-carrying running and aerobic conditioning, combined with strength training, could undoubtedly be a highly effective solution to significantly mitigate muscle damage and fatigue in men and women. This approach would benefit both the upper and lower back muscles responsible for accommodating and stabilizing the weight carried on the shoulders by the backpack and plate carrier, as well as the muscles involved in running, which are essential for absorbing impact and propelling both body mass and additional load with each stride [59]. However, without proper training periodization, achieving the right balance between training stimulus stress and recovery can be challenging, potentially leading to military personnel injuries and resulting in personal and economic resource losses for the armed forces.

The primary limitation of this study was the inability to standardize food and fluid intake before, during, and after the test to homogenize dietary consumption. Additionally, the sample size may have been significantly reduced due to dropout rates caused by injury or illness, as the test administered was physically demanding. Therefore, special attention must be given to the recovery and the management training loads for military personnel in the days following such tests due to the high levels of fatigue they induce, especially if additional common military exercises are required within a short period of time. In this regard, the role of the tactical strength and conditioning instructor is crucial.

## 5. Conclusions

In conclusion, based on the observed data, the results indicated that the test had a significant neuromuscular impact, with values remaining below baseline levels at 48 h. This is especially important during maneuvers or military exercises in operational units, where numerous training exercises are frequently conducted under stressful conditions. Each of the tests conducted aims to assess performance in various aspects relevant to the military profession (such as endurance, muscular strength, power, coordination, etc.), as these data will allow us to understand to what extent a test of this nature affects military personnel in the tasks inherent to their profession that they may need to perform in the following days, such as jumping or clearing obstacles, moving with agility and coordination, carrying and dragging heavy loads, running long distances, and more [5]. With advancements in sports technology, regular monitoring of neuromuscular performance, as well as before and after highly demanding tests, could be a valid strategy to assess performance status, track recovery progress, and ensure optimal neuromuscular adaptations.

## Data Availability

The datasets used and/or analyzed during the current study are available from the corresponding author upon reasonable request.

## Appendix A

### Appendix A.1

**Table A1:** Performance variable results at different time points.

	PRE	POST	24 H POST	48 H POST	ES ^1^	ES ^2^	ES ^3^
ROF [points]	1.96 ± 0.56	7.57 ± 0.66 ‡	4.96 ± 1.19 ‡Ψ	3.43 ± 1.12 ‡ΨͲ	−6.29	−2.12	−1.10
BM [kg]	80.00 ± 7.79	77.26 ± 7.88 ‡	78.40 ± 7.81 ‡¥	78.55 ± 8.03 ‡Ψ	1.88	2.50	2.06
PUmax [reps]	17.70 ± 3.23	13.91 ± 3.48 ‡	15.65 ± 2.72 ‡Ψ	16.65 ± 3.19 ‡ΨͲ	2.01	1.54	1.48
DHGS [kg]	49.71 ± 6.86	51.32 ± 7.38	51.21 ± 7.33	50.30 ± 6.94	−0.43	−0.35	−0.14
NDHGS [kg]	47.15 ± 7.05	48.65 ± 6.72	47.71 ± 6.31	47.90 ± 6.34	−0.22	−0.12	−0.12
IMTP [N]	2612.26 ± 290.99	2636.70 ± 292.59	2463.43 ± 301.28 †§	2486.43 ± 247.72 ‡§	−0.09	0.61	0.79
VJ CMJ [cm]	32.16 ± 5.96	27.78 ± 6.23 ‡	28.47 ± 4.81 ‡	27.67 ± 4.50 ‡	1.16	1.27	1.20
PP CMJ [W]	3741.83 ± 573.54	3703.17 ± 499.01	3420.83 ± 417.86 †¥	3401.26 ± 462.75 ‡¥	0.08	0.65	0.76
RFDEcc CMJ [N/s]	3469.65 ± 2562.48	3318.61 ± 1971.24	3695.43 ± 1978.30	3592.96 ± 2044.50	0.11	−0.16	−0.08
RFDCon CMJ [N/s]	1995.13 ± 1756.84	2123.52 ± 1509.48	1077.99 ± 935.26 †¥	1754.91 ± 1421.64 Ᵹ	−0.05	0.59	0.14
PEcc CMJ [N]	1605.13 ± 362.37	1494.83 ± 370.90 *	1598.30 ± 315.69 Ψ	1611.22 ± 315.12 ¥	0.51	0.03	−0.03
PCon CMJ [N]	1775.61 ± 250.79	1805.57 ± 197.93	1735.61 ± 217.53	1781.48 ± 231.25	−0.15	0.27	−0.05

BM: Body Mass; CMJ: countermovement jump; DHGS: Dominant hand grip strength; ES ^1^: Effect size pre-post; ES ^2^: Effect size pre-24 h post; ES ^3^: Effect size pre-48 h post; IMTP: Isometric mid-thigh pull; NDHGS: Non-dominant hand grip strength; PCon: Peak concentric force; PEcc: Peak eccentric force; PP: Peak power; RFD: Rate of force development; ROF: Rating of fatigue scale. VJ: Maximum vertical jump height from take-off; Values are represented as means ± SD. The following symbols indicate significant differences relative to (a) pre: * = significant difference (*p* ≤ 0.05); † = significant difference (*p* ≤ 0.01); ‡ = significant difference (*p* ≤ 0.001); (b) Post: § = significant difference (*p* ≤ 0.05); ¥ = significant difference (*p* ≤ 0.01); Ψ = significant difference (*p* ≤ 0.001); and (c) 24 h post: Ᵹ = significant difference (*p* ≤ 0.05); Ͳ = significant difference (*p* ≤ 0.001).

### Appendix A.7

**Table A2:** Report on incidents and perception of effort following 30 K loaded carriage.

	T1 (0–10 km)	T2 (10–15 km)	T3 (15–20 km)	T4 (20–25 km)	T5 (25–30 km)	ES ^1^	ES ^2^	ES ^3^	ES ^4^
Fatigue	30.00 ± 22.16	32.61 ± 22.00	37.83 ± 25.93 §	41.74 ± 26.74 †¥	54.78 ± 29.52 ‡ΨͲΣ	−0.15	−0.38	−0.60	−0.99
Muscle pain	37.83 ± 24.30	39.13 ± 24.66	53.48 ± 23.86 †Ψ	61.30 ± 24.74 ‡ΨꝽ	70.43 ± 19.42 ‡ΨͲꝊ	−0.06	−0.66	−1.31	−1.48
Joint pain	33.91 ± 26.07	32.61 ± 22.00	40.43 ± 26.54 §	49.57 ± 29.46 ‡ΨꝽ	60.00 ± 28.60 ‡ΨͲΣ	0.06	−0.32	−1.02	−1.39
Shortness of breath	24.78 ± 14.73	24.78 ± 14.73	24.78 ± 14.73	30.00 ± 20.23 *§Ᵹ	35.22 ± 23.33 †¥ŦꝊ	ND	ND	−0.45	−0.71
Excessive sweating	27.39 ± 17.89	31.30 ± 19.14	31.30 ± 16.87	41.74 ± 25.16 †¥Ŧ	48.26 ± 25.16 ‡ΨͲ	−0.38	−0.29	−0.72	−0.91
Muscle tremor	24.78 ± 14.73	23.48 ± 12.65	24.78 ± 14.73	26.09 ± 16.44	30.00 ± 18.09 §Ᵹ	0.21	0.00	−0.12	−0.35
Palpitations	24.78 ± 14.73	26.09 ± 16.44	26.09 ± 16.44	26.09 ± 16.44	30.00 ± 20.23	−0.12	−0.12	−0.12	−0.30

ES ^1^: Effect Size T1–T2; ES ^2^: Effect Size T1–T3; ES ^3^: Effect Size T1–T4; ES^4^: Effect Size T1–T5; ND: Not Determined. Values are represented as means ± SD. The following symbols indicate significant differences relative to (a) 0–10 km: * = significant difference (*p* ≤ 0.05); † = significant difference (*p* ≤ 0.01); ‡ = significant difference (*p* ≤ 0.001); (b) 10–15 km: § = significant difference (*p* ≤ 0.05); ¥ = significant difference (*p* ≤ 0.01); Ψ = significant difference (*p* ≤ 0.001); (c) 15–20 km: Ᵹ = significant difference (*p* ≤ 0.05); Ŧ = significant difference (*p* ≤ 0.01); Ͳ = significant difference (*p* ≤ 0.001); and (d) 20–25 km: Ꝋ = significant difference (*p* ≤ 0.05); Σ = significant difference (*p* ≤ 0.01).
